# CRISPR RNA-guided *Fok*I nucleases repair a *PAH* variant in a phenylketonuria model

**DOI:** 10.1038/srep35794

**Published:** 2016-10-27

**Authors:** Yi Pan, Nan Shen, Sabine Jung-Klawitter, Christian Betzen, Georg F. Hoffmann, Jörg D. Hoheisel, Nenad Blau

**Affiliations:** 1 Division of Functional Genome Analysis, German Cancer Research Center (DKFZ), Heidelberg, Germany; 2 Dietmar-Hopp-Metabolic Center, Department of General Pediatrics, University Hospital, Heidelberg, Germany; 3 Department of General Pediatrics I, University Hospital, Heidelberg, Germany; 4 University Children’s Hospital, Zürich, Switzerland

## Abstract

The CRISPR/Cas9 system is a recently developed genome editing technique. In this study, we used a modified CRISPR system, which employs the fusion of inactive Cas9 (dCas9) and the *Fok*I endonuclease (*Fok*I-dCas9) to correct the most common variant (allele frequency 21.4%) in the phenylalanine hydroxylase (*PAH*) gene - c.1222C>T (p.Arg408Trp) - as an approach toward curing phenylketonuria (PKU). PKU is the most common inherited diseases in amino acid metabolism. It leads to severe neurological and neuropsychological symptoms if untreated or late diagnosed. Correction of the disease-causing variants could rescue residual PAH activity and restore normal function. Co-expression of a single guide RNA plasmid, a *Fok*I-dCas9-zsGreen1 plasmid, and the presence of a single-stranded oligodeoxynucleotide in *PAH*_c.1222C>T COS-7 cells – an *in vitro* model for PKU – corrected the *PAH* variant and restored PAH activity. Also in this system, the HDR enhancer RS-1 improved correction efficiency. This proof-of-concept indicates the potential of the *Fok*I-dCas9 system for precision medicine, in particular for targeting PKU and other monogenic metabolic diseases.

Phenylketonuria (PKU, OMIM 261600) is the most frequent and important inherited metabolic disease, caused by deficiency of hepatic phenylalanine hydroxylase (PAH, EC 1.14.16.1). PAH converts phenylalanine (Phe) to tyrosine (Tyr). The cofactor tetrahydrobiopterin (BH_4_), iron and molecular oxygen are required for this process^1^. PAH deficiency results in the accumulation of Phe in the body including the brain. If untreated or diagnosed late, its high concentration leads to severe neurological and neuropsychological symptoms, including intellectual disability, seizures, ataxia, motor deficits and behavioral problems. Because of the severity of the disease, newborn screening for PKU has been instituted in many countries. Lifelong restriction of dietary Phe is the established treatment for preventing neuronal damage. However, poor compliance by PKU patients and the heavy economic burden challenge the dietary management. Although the restriction of dietary Phe improves cognitive performance, other neuropsychological parameters such as choice reaction time, attention, and executive functions are impaired compared to healthy controls even in the best treated individuals. An additional supplement of tetrahydrobiopterin (BH_4_; sapropterin dihydrochloride) may be beneficial for some PKU patients with a mild pheotype^2^. However, the ideal approach for curing PKU would be to correct variants in the *PAH* gene directly through gene therapy. Since PKU is an autosomal recessive inherited disorder, only individuals with *PAH* variants located in both alleles develop symptoms. Even if only one allele is corrected, the residual PAH activity would be sufficiently increased, abolishing or reducing PKU symptoms. In patients with severe classic PKU, the c.1222C>T (p.Arg408Trp) single nucleotide variant is the most frequent one in *PAH* (allele frequency 21.4%) and p.[Arg408Trp];[Arg408Trp] the most common genotype (genotype frequency 10.2%)^3^. Thus, *PAH*_c.1222C>T was chosen as the target in this study.

The most promising genetic repair of variants is achievable through homologous recombination repair (homology directed repair; HDR) after the introduction of DNA double-stranded breaks (DSBs) close to the variant. DSBs can be generated by different genome editing technologies, such as zinc-finger nucleases (ZFNs), transcription activator-like effector nucleases (TALENs) and the recently developed technique of clustered regularly interspaced short palindromic repeats (CRISPR)^4^. The CRISPR/Cas9 system was first identified as a prokaryotic defense mechanism against bacteriophages, and is currently being used for site-specific genome editing. It is composed of a single-guide RNA (sgRNA) containing a 20 nt sequence that targets a specific genomic site and one Cas9 nuclease that enables the cleavage of genomic DNA. The DSBs then stimulate the DNA repair pathway through non-homologous end joining (NHEJ) and HDR. Of these mechanisms, HDR can generate the desired sequence replacement at the DSBs through the usage of a donor DNA template, which corrects the specific variant within the genome^5^. Several studies have recently reported encouraging results using CRISPR/Cas9 for successful therapeutic concepts, such as Duchenne muscular dystrophy and retinitis pigmentosa *in vitro* and *in vivo*^6^,^7^,^8^,^9^,^10^. However, there are still two major challenges in the CRISPR/Cas9-mediated genome correction: frequent off-target effects and low HDR efficiency^5^,^11^,^12^. To overcome these problems, different strategies were adopted in this study. First, we chose the CRISPR RNA-guided *Fok*I nuclease system (*Fok*I-dCas9 system)^13^,^14^,^15^ instead of the CRISPR/Cas9 system as the specific genome editing tool. The *Fok*I-dCas9 system is composed of two distinct sgRNAs and *Fok*I-dCas9 nuclease, which is a fusion of inactive Cas9 (dCas9) and the *Fok*I endonuclease. The *Fok*I-dCas9 system differs from CRISPR/Cas9 in that the simultaneous binding of two distinct *Fok*I-dCas9:sgRNAs is required. Moreover, we employed 3-(N-benzylsulfamoyl)-4-97 bromo-N-(4-bromophenyl)benzamide (RS-1), an HDR enhancer^16^,^17^, as a RAD51-stimulatory compound^18^. By this means, we successfully performed a genetic repair of the c.1222C>T *PAH* variant, which for the first time corrected the point variant and rescued both PAH protein expression and activity.

## Results

### Establishment of the *PAH*_c.1222C>T COS-7 cell line as an *in vitro* PKU model

**Figure 1:**
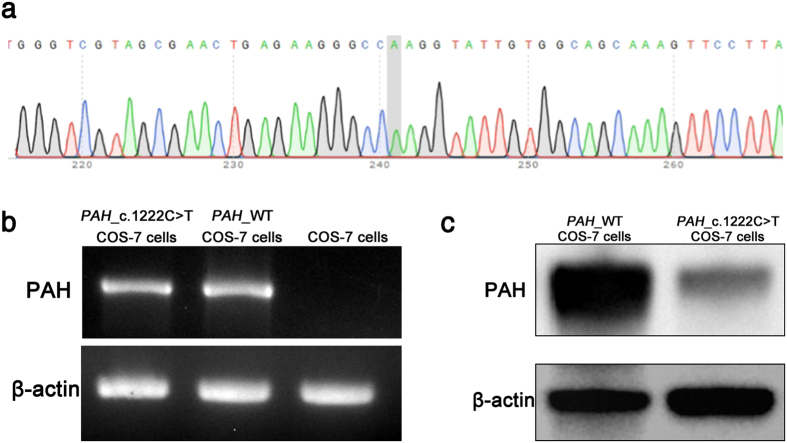
*PAH*_c.1222C>T COS-7 cell line was established as a PKU *in vitro* model. (**a**) Sanger sequencing analysis of *PAH*_c.1222C>T COS-7 cells by IRES reverse primer showed “A” point mutation on the PAH gene. (**b**) RT-PCR confirmed *PAH*_c.1222C>T expression in established cell line. (**c**) Western blot showed that the expression of PAH in *PAH*_c.1222C>T cells was lower than in *PAH*_WT cells.

The *PAH*_c.1222C>T variant was introduced into COS-7 cells by transfection with an appropriately modified vector DNA. Single clones were sub-cultured for at least 15 passages. Sanger sequencing analysis proved that the “T” point variant was appropriately located in *PAH* (Fig. 1a). Reverse transcription PCR (RT-PCR) confirmed that *PAH*_c.1222C>T was expressed in the transfected COS-7 cells at the RNA level at a similar level as the wild-type protein used in control; untransfected COS-7 cells exhibited no *PAH* RNA (Fig. 1b). Concurrently, Western blot analysis with an antibody against the wild-type protein (PAH_WT) revealed a basically complete loss of the PAH protein in the cells transfected with the mutated gene version (Fig. 1c).

### The *PAH*_c.1222C>T variant was corrected using the *Fok*I-dCas9 system

**Figure 2:**
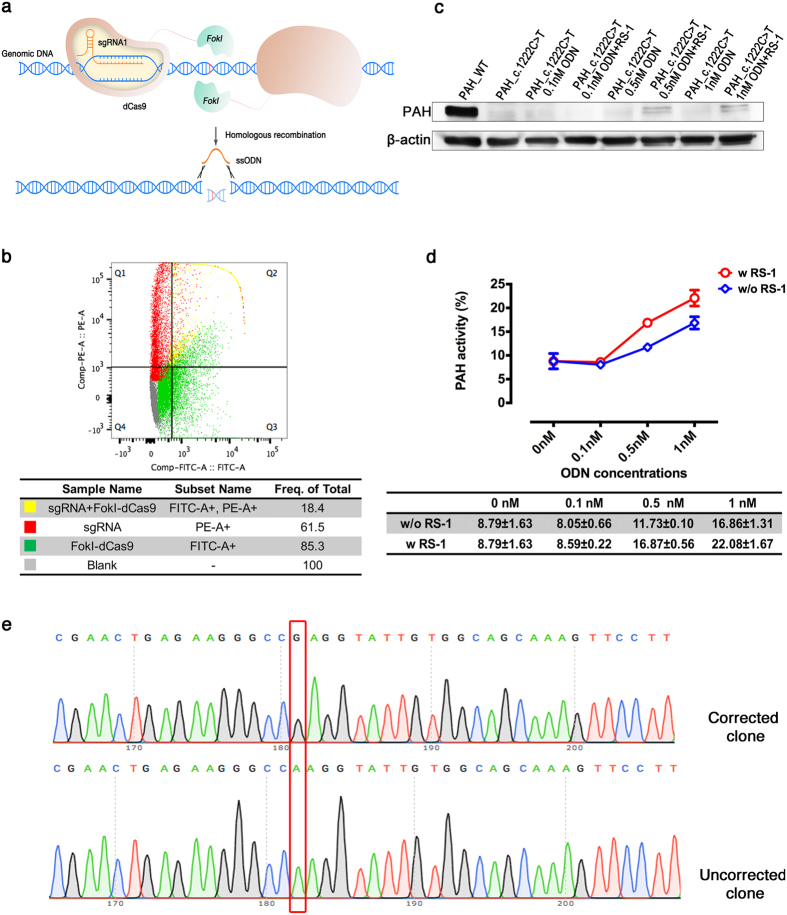
Correction of the *PAH*_c.1222C>T variant in COS-7 cells. (**a**) Scheme of the process: one dimer of the *Fok*I-dCas9 complex binds to two “half-sites” on the genome with a certain spacer length and generate DSBs. DSBs are then repaired by HDR. (**b**) Co-expression efficiency of the *Fok*I-dCas9 and sgRNA plasmids in *PAH*_c.1222C>T COS-7 cells was analyzed by flow cytometry. (**c**) The PAH expression of each cell population was detected by Western blot. (**d**) The PAH activity of each group was measured by liquid chromatography-electrospray ionization tandem mass spectrometry. (**e**) Correction of variant; one of 30 sequences are shown. T/A-cloning into *E. coli* and Sanger sequencing were performed on DNA isolated from individual COS-7 cells transfected with the *Fok*I-dCas9 and sgRNA plasmids as well as 1 nM ssODN and treated with RS-1. Among all 30 randomly picked colonies, representing the DNA of 30 COS-7 cells, 26.7% of the cells exhibited correction of the variant.

The *Fok*I-dCas9 system is different from the CRISPR/Cas9 system insofar as only binding of a *Fok*I-dCas9 dimer can cleave DNA (Fig. 2a). After electroporation of COS-7 cells with expression vectors of the *Fok*I-dCas9 gene and the sgRNA, flow cytometry analysis showed that approximately 18.4% of cells co-expressed them simultaneously (Fig. 2b). In presence of RS-1, PAH expression in *PAH*_c.1222C>T COS-7 cells treated with 0.5 or 1 nM of the single-stranded oligodeoxynucleotide (ssODN) that represents the replacement sequence was significantly higher than the PAH expression of *PAH*_c.1222C>T COS-7 cells that were not transfected with the vectors. A lower ssODN concentration had no apparent effect on PAH expression (Fig. 2c). Furthermore, liquid chromatography-electrospray ionization tandem mass spectrometry was employed to evaluate the PAH activity after repair (Fig. 2d), confirming the results of the Western blot analysis. Sanger sequencing analysis was performed to confirm the correction in all groups. The 1 nM ssODN and 0.5 nM ssODN groups showed double-peaks at the corrected position regardless of RS-1 treatment, demonstrating the presence of both a “T” and a “C” at the *PAH*_c.1222C>T variant in the cell population (Supplementary Fig. 1a). To examine the correction rate, we amplified by PCR target DNA from the 1 nM ssODN with RS-1 treatment group and verified the sequence by TA-cloning. Sequence analysis of the DNA of 30 colonies showed that 8 of them (26.7%) contained the correct “C” (Fig. 2e). To assess the possible off-targeting introduced by *Fok*I-dCas9-directed cleavage, two potential off-target sites were predicted by CasOT (Supplementary Table 1). Both potential sites were analyzed together with the *PAH* gene on-target site by Sanger sequencing. None of the analyzed regions showed evidence of off-target cleavage (data not shown).

## Discussion

Although the restriction of dietary Phe and/or supplementation with BH_4_ can substantially alleviate clinical symptoms of PKU, patients do not manage to maintain dietary treatment for life. They suffer from impaired neuropsychological and partly from intellectual function and PKU women are at risk for the maternal PKU syndrome. The ideal approach to treating this disease would be a correction of the *PAH* variant. PKU gene therapy has been developed over the past two decades using an animal model. Researchers delivered the wild-type *PAH* gene to tissues with an adeno-associated virus (AAV) vector. However, the temporary recovery of PAH activity, risks associated with the AAV vector or a very low gene transfer rate in the past limited the development of this treatment option^19^,^20^. Ongoing research is focused on new genome editing technologies that can correct genomic variants. Therefore, in this study the *Fok*I-dCas9 system was chosen to correct with high accuracy and efficiency the most common *PAH* variant in cells.

The CRISPR/Cas9 system stimulates DNA repair mechanisms such as NHEJ and HDR. NHEJ leads to random deletion or insertion variants close to the DSB site, resulting in gene knock out, while HDR can generate a desired sequence replacement at the DSB by donating a DNA template, resulting in gene correction. Despite the tremendous potential of the original CRISPR/Cas9 system^21^, several challenges still limit its application to gene therapy. For example, the short 20 bp target sequence required in the sgRNA, of the commonly used SpCas9 system may cause off-target effects elsewhere in the genome^12^. Moreover, NHEJ is the dominant mechanism for repairing DSBs after DNA cleavage in mammals, preventing gene correction by HDR. Thus, new strategies that avoid off-target effects and increase the HDR:NHEJ ratio are needed^22^.

The *Fok*I-dCas9 system can greatly improve the specificity of genome editing *in vitro* and *in vivo.* In contrast to the CRISPR/Cas9 system, it requires dimerization of the *Fok*I-dCas9-sgRNA complex, meaning that monomeric *Fok*I-dCas9-sgRNA is unable to cut the DNA strand^14^. Three rules are used in designing a specific *Fok*I-dCas9-sgRNA complex that retains high cleavage efficiency. First, the pair of sgRNAs should bind to sense and antisense strands. Second, two distinct *Fok*I-dCas9-sgRNA-binding “half-sites” must be located close to the targeted genomic position, and third, the *Fok*I-dCas9-sgRNA complex requires a particular orientation. Generally, having protospacer adjacent motif (PAM) sequences for the two distinct *Fok*I-dCas9-sgRNA complexes located on the outer boundaries of the target site (“PAM-out” orientation) is superior to having them connected directly to the spacer sequence (“PAM-in” orientation). In addition, the spacer length between two PAM sequences has limitations. Fourteen to 17 bp of spacer length exhibit higher on-target cleavage efficiency than other spacer lengths between 0 to 31 bp^15^. Based on these rules, this study chose the *Fok*I-dCas9-sgRNA complex with a “PAM-out” orientation and spacer length of 17 bp. Moreover, it is worth noting that while a single *Fok*I-dCas9-sgRNA can bind elsewhere in the genome, it cannot cleave DNA and cause off-target effects. In addition, *Fok*I-dCas9 targets DNA sites with more than 140-fold higher specificity than normal SpCas9 and higher specificity than Cas9 nickases, which only cut single DNA strands throughout the genome^23^.

Although besides *Fok*I-dCas9, strategies like Cas9 nickases or high-fidelity eSpCas9 variants^24^,^25^ can be adopted to enhance Cas9 specificity, we selected the FokI-dCas9 system to correct the PAH variant for its dual advantage of high specificity^14^ and potential improvement of HDR efficiency by DNA template preservation. It is generally known that the generation of HDR requires a large amount of DNA template. In the CRISPR/Cas9, eSpCas9 or Cas9 nickases systems, the DNA template can be cut by nucleases, even if an ssODN is used as the repair template. However, the *Fok*I-dCas9-sgRNA complex cannot cleave the ssODN template as two distinct *Fok*I-dCas9-sgRNA complexes have to bind to different DNA strands. Therefore, the CRISPR RNA-guided *Fok*I nucleases system ensures a rich source of repair template during the HDR process. To further enhance HDR efficiency, a small-molecule drug was used in our study. The potent NHEJ inhibitor 5,6-bis((E)-benzylideneamino)-2- mercaptopyrimidin-4-ol (SCR-7) and the HDR enhancer RS-1, can be used to improve nuclease-mediated HDR efficiency^16^,^26^. RS-1 shows greater potential than SCR-7 in improving the HDR rate *in vivo*^17^. Our study found that, after electroporation with the *Fok*I-dCas9-sgRNA complex, the *PAH*_c.1222C>T COS-7 cells remaining in RS-1 culture medium showed more than twice as much PAH activity as negative controls. Also, the PAH expression of the RS-1 group was significantly higher than cells grown without RS-1. The 26.7% correction rate can be expected to provide a clinically sufficient therapeutic effect, as normal development has been reported in patients with PAH activities as low as 8.7–34.5% of normal^27^. RS-1 can increase knock-in efficiency by two- to five-fold in both the TALEN- and Cas9-mediated knock-in systems^17^. We found a similar improvement in HDR efficiency in the *Fok*I-dCas9 system.

Using LC-ESI-MS-MS, we could determine in *PAH*_c.1222C>T COS-7 cells electroporated with the sgRNA plasmid, *Fok*I-dCas9 plasmid and 1 nM ssODN and cultured in RS-1 a PAH activity of 22.1% compared to 8.8% in the control group. The goal of this study was not only the genetic repair of the *PAH* variant, but also the rescue of PAH activity after correction. The rescue of PAH activity demonstrates that our strategy is a promising approach for PKU treatment.

In conclusion, this study indicates that the *Fok*I-dCas9 system is a suitable, specific and effective genome editing technique for correcting a major *PAH* variant that causes PKU. This highlights its tremendous potential for the precise treatment of also other inherited metabolic diseases.

## Methods

### Construction of the *PAH*_c.1222C>T vector and establishment of the *PAH*_c.1222C>T COS-7 cell line

A wild-type *PAH* cDNA was cloned into the pLVX-EF1α-IRES-Puro vector (Clontech) using *Xba*l and *BamH*I cleavage (Thermo Fisher Scientific) after the amplification of cDNA from the pCMV-FLAG-*PAH* wild-type plasmid. The *PAH*_c.1222C>T variant in the *PAH* cDNA sequence was introduced by site-directed mutagenesis using the QuikChange II XL Site-Directed Mutagenesis Kit (Agilent Technologies) and confirmed by Sanger DNA sequencing (GATC Biotech). EF1α forward and IRES reverse sequencing primers (Thermo Fisher Scientific) were used to sequence the *PAH*_c.1222C>T variant.

COS-7 cells were cultured at 37 °C with 5% CO_2_ in Dulbecco’s Modified Eagle Medium (Thermo Fisher Scientific) supplemented with 10% fetal bovine serum and 1% penicillin/streptomycin. One day prior to transfection, 2 × 10^6^ cells were seeded and cultured in one dish of 10 cm diameter. The transfection of one dish utilized 45 μL Fugene HD reagent (Promega) and 15 μg *PAH*_c.1222C>T plasmid or the plasmid with the wild-type sequence as a control. Forty-eight hours post transfection, the growth medium was replaced with complete growth medium containing 10 μg/mL puromycin as the selective agent. After the non-transfected COS-7 cells had died, a single *PAH*_c.1222C>T COS-7 clone was isolated using limiting dilution cloning technique performed in 96-well plates with 10 μg/mL puromycin remaining in the culture medium. The clone was characterized through RT-PCR, and expanded in 75 cm^2^ cell culture flasks.

### Construction of the CRISPR RNA-guided *Fok*I system plasmids and the HDR donor

The IRES-ZsGreen1 fragment of the pLVX-EF1α-IRES-ZsGreen1 vector (Clontech, Mountain View, USA), was used to construct by means of the Megaprimer PCR method^28^ the *Fok*I-dCas9-IRES-ZsGreen1 plasmid (Supplementary Fig. 1b).

gRNAs, targeting the *PAH*_c.1222C>T variant were designed with the E-CRISP software tool (www.e-crisp.org)^29^. One suitable gRNA pair, which is composed of two separate gRNAs, was chosen as previously described^15^. gRNA pairs should be located on either side of the target in the PAM-out orientation and contain spacer of 14–17 bp in length. The final sgRNA expression fragment (Supplementary Table 2) was synthesized using gBlocks (Integrated DNA Technologies). It contains two separate U6 promoters, gRNAs and tracrRNAs, but is free of any 5′ modifications. The synthesized sgRNA fragment was cloned into the pRSI9 vector (Cellecta) using Gibson Assembly Cloning (New England Biolabs) resulting in the pRSI9-1222sgRNA plasmid (Supplementary Fig. 1c).

The ssODN was synthesized using an HDR template from Integrated DNA Technologies. The homology arm of the HDR template located on the 5′end of the *PAH*_c.1222C>T variant position is 71 nt and the arm on the 3′end is 111nt (Supplementary Table 2).

### Electroporation of *PAH*_c.1222C>T COS-7 cells

Electroporation was performed according to the instructions provided with the Gene Pulse Xcell electroporation system (Bio-Rad). Prior to electroporation, *PAH*_c.1222C>T COS-7 cells were washed with PBS and resuspended in Gene Pulser electroporation buffer (Bio-Rad) at 2.5 × 10^6^ cells/mL. Ten micrograms of pRSI9-1222sgRNA plasmid, 10 μg *Fok*I-dCas9-zsGreen plasmid and 0.1 nM to 1 nM ssODN were added to the 0.4 cm cuvette with 400 μL cells and mixed gently. The optimized electroporation setting for *PAH*_c.1222C>T COS-7 cells was previously determined to be the following: pulse type (square wave), V (200 V), PL (15 msec) and cuvette (0.4 cm). After electroporation, 400 μL of the electroporated cells were transferred into 6-well plates. These electroporated cells were cultured in complete growth medium with or without 15 μM RS-1 (Sigma-Aldrich) at 37 °C for 96 hours.

### Flow cytometry analysis

Ninety-six hours after electroporation, cell pellets were harvested by centrifugation at 200 g for 4 min and resuspended in PBS buffer to 0.5 × 10^6^ cells/mL. Flow cytometry analysis was performed on a BD FACSVerse™ flow cytometer (BD Biosciences). *PAH*_c.1222C>T COS-7 cells containing the 1222sgRNA plasmid were identified as PE positive while cells transfected with the *Fok*I-dCas9-zsGreen plasmid were FITC positive. When the 1222sgRNA and *Fok*I-dCas9-zsGreen plasmids were co-expressed, the cell group was gated as both PE and FITC positive group. The data were acquired and analyzed with FLOWJO software (FLOWJO).

### Measurement of PAH activity by liquid chromatography-electrospray ionization tandem mass spectrometry

The PAH activity of the cell lysates was measured using previously described methods^30^. In brief, 5 μL (containing 5–10 μg of total protein) of cell homogenate obtained from *PAH*_c.1222C>T COS-7 cells after electroporation was mixed with 0.1 M Na-HEPES buffer, 2 μg catalase and 1 M L-Phe and incubated for 5 min. One μM Fe(NH_4_)_2_(SO_4_)_2_ was added into the mixture for one minute. PAH assay reaction was started by adding 200 μM BH_4_ and then incubated at 25 °C for 15 min. The reaction was stopped by adding 50 μL of 2% (w/v) acetic acid in ethanol. Then, the PAH samples were prepared for liquid chromatography-electrospray ionization tandem mass spectrometry in accordance with the EZ:faast^TM^ kit manual (Phenomenex). Ten microliters of 100 μM Phe-d_5_ and 10 μL of 20 μM Tyr-d_4_ were added to 80 μL PAH assay samples. The Phe and Tyr concentrations were calculated from an internal standard ratio. The specific PAH activity was expressed as mU/mg total protein, with mU equal to nmol Tyr produced. The PAH activity of *PAH*_wild-type COS-7 cells was used as a positive control and set at 100%.

### Immunoquantification by Western blot analysis

Small amounts (10–15 μg) of protein lysate from the PAH activity assay were used to detect PAH expression by Western blot. An anti-PAH antibody (Merck Millipore) and an anti-β-actin antibody (Santa Cruz Biotechnology) were used as primary antibodies at a 1:5,000 dilution and 1:2,000 dilution, respectively. A goat anti-mouse IgG-HRP conjugated to horseradish peroxidase (Santa Cruz Biotechnology) was used as the secondary antibody for detection at a 1:10,000 dilution. Signals were generated and recorded using the SuperSignal West Pico Chemiluminescent Substrate (Thermo Fisher Scientific) as described by the manufacturer’s instructions.

### TA cloning and sequencing of the *PAH*_c.1222C>T variant reversal

Genomic DNA from electroporated *PAH*_c.1222C>T COS-7 cells was extracted using NucleoSpin Tissue (Macherey-Nagel) according to the manufacturer’s instructions. PCR amplification of the mutated target sites was performed with Q5 High-Fidelity DNA Polymerase (New England Biolabs) at standard conditions. The PCR product was purified with the PureLink PCR Purification Kit (Thermo Fisher Scientific). Poly-A tailing of the purified DNA was completed using Taq Polymerase (Qiagen), followed by cloning into plasmid pMD19-T (Clontech). Upon transformation into *E. coli* cells and colony growth, DNA was isolated from individual colonies and analyzed by Sanger sequencing, using EF1α forward and IRES reverse sequencing primers.

### Sanger sequencing for off-target analysis

The potential off-target sites have been predicted using CasOT (http://eendb.zfgenetics.org/casot/)^31^, a genome-wide potential Cas9-gRNA off-target searching online tool. The genomic region surrounding the candidate off-target sites were PCR-amplified with Q5 High-Fidelity DNA Polymerase (New England Biolabs) at standard conditions, and the PCR products were purified using PureLink PCR Purification Kit (Thermo Fisher Scientific). Sanger sequencing was utilized to detect their sequences using primers which covered the regions of these sites.

## Additional Information

**How to cite this article**: Pan, Y. *et al*. CRISPR RNA-guided *Fok*l nucleases repair a *PAH* variant in a phenylketonuria model. *Sci. Rep.* **6**, 35794; doi: 10.1038/srep35794 (2016).

**Publisher’s note:** Springer Nature remains neutral with regard to jurisdictional claims in published maps and institutional affiliations.

## Supplementary Material

Supplementary Information
