# Supplementary material for: CRISPR RNA-guided FokI nucleases repair a PAH variant in a phenylketonuria model
Source: Sci Rep. 2016 Oct 27;6:35794. doi: 10.1038/srep35794 (PMC5081532; doi:10.1038/srep35794)

CRISPR RNA-guided *Fok*I nucleases repair a *PAH* variant in a phenylketonuria model

Yi Pan, Nan Shen, Sabine Klawitter-Jung, Christian Betzen, Georg F Hoffmann, Jörg D Hoheisel, Nenad Blau

Supplementary Figure 1. COS-7 cell line with *PAH*_c.1222C>T mutation was corrected by dCas9-FokI. [a] Sanger sequencing of DNA after correction showed a minor “G” peak at the same position, indicating a partial correction of the C>T variant in PAH_c.1222C>T COS-7 cells. [b]The maps of FokI-dCas9-IRES-ZsGreen1 plasmid and [c]pRSI9-U6-sgRNAs plasmid.

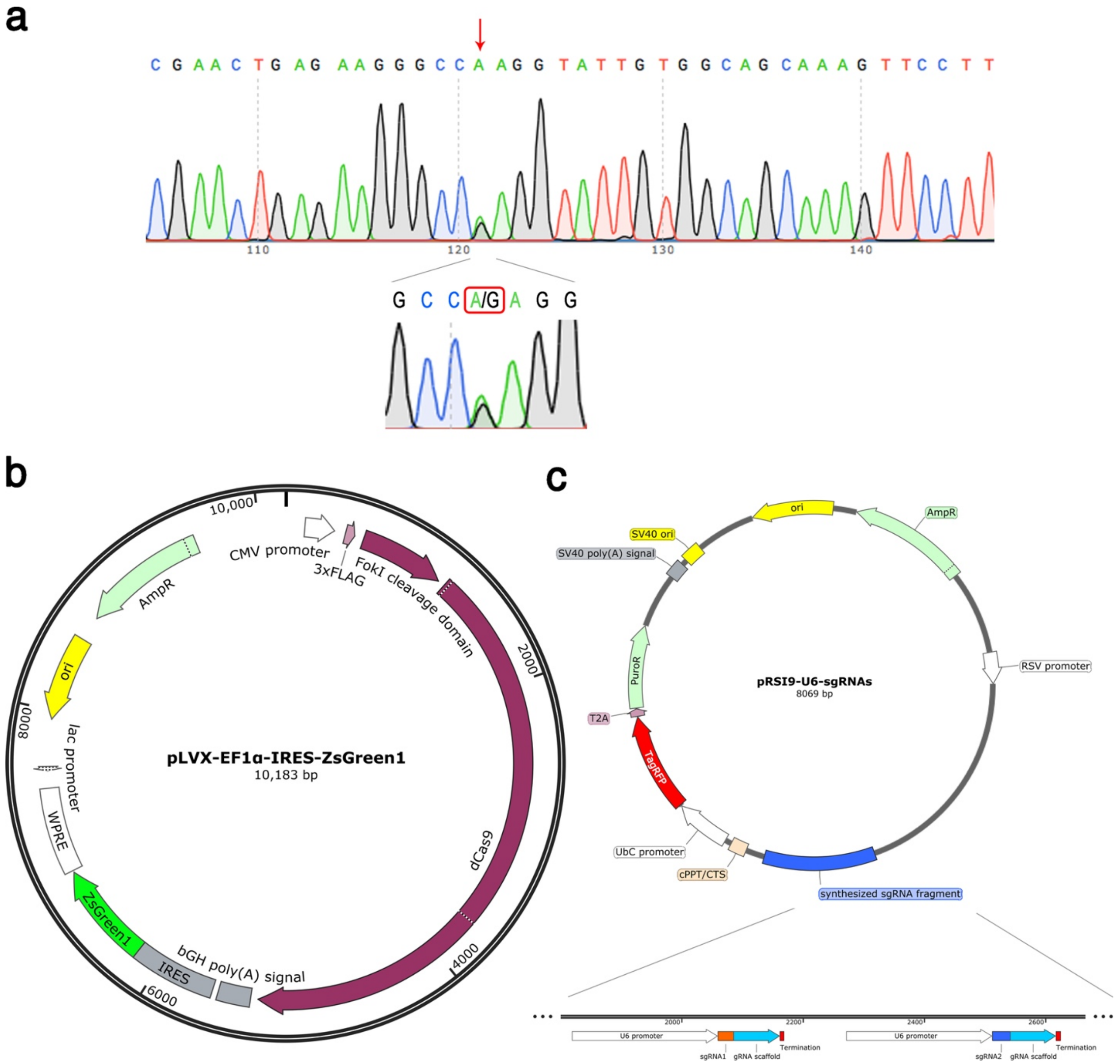

CRISPR RNA-guided *Fok*I nucleases repair a *PAH* variant in a phenylketonuria model

Yi Pan, Nan Shen, Sabine Klawitter-Jung, Christian Betzen, Georg F Hoffmann, Jörg D Hoheisel, Nenad Blau

Supplementary Table 1. The potential off-target sites have been predicted using CasOT.

Potential Target	Spacer Length	Sequence
NC_023652.1: 98067933-98067997	17	TCCAAGACCTCAATCCTTTGGGTGTATGGGTCATAGCGA ACTGAGAAGGGCCGAGGGATTGTGGC
NC_023654.1: 97328309-97328378	22	TCCAAGACCTGAATCTATGAAACTACTAGAAGAATACAT TGGGCAAGTGCTCAAGGATTTTGGTCTGGGC

CRISPR RNA-guided *Fok*I nucleases repair a *PAH* variant in a phenylketonuria model

Yi Pan, Nan Shen, Sabine Klawitter-Jung, Christian Betzen, Georg F Hoffmann, Jörg D Hoheisel, Nenad Blau

Supplementary Table 2. sgRNAs and ssODN synthesized sequences for the CRISPR RNA-guided FokI nucleases to genetic repair of *PAH*_c.1222C>T. The text in green shows gRNA sequences. The character in red shows the correction site.

**sgRNAs**	TCTTGTGGAAAGGACGAAACGTGAGAAGGGCCAAGGTATTGGTTTTAGAGCTAGAAATAGCA<br>AGTTAAAATAAGGCTAGTCCGTTATCAACTTGAAAAAGTGGCACCGAGTCGGTGCTTTTTTTCT<br>AGACCCAGCTTTCTTGTACAAAGTTGGCATTATGTACAAAAAAGCAGGCTTTAAAGGAACCAA<br>TTCAGTCGACTGGATCCGGTACCAAGGTCGGGCAGGAAGAGGGCCTATTTCCCATGATTCCTT<br>CATATTTGCATATACGATACAAGGCTGTTAGAGAGATAATTAGAATTAATTTGACTGTAAACACA<br>AAGATATTAGTACAAAATACGTGACGTAGAAAGTAATAATTTCTTGGGTAGTTTGCAGTTTTAA<br>AATTATGTTTTAAAATGGACTATCATATGCTTACCGTAACTTGAAAGTATTTCGATTTCTTGGCTT<br>TATATATCTTGTGGAAAGGACGAAACACCGCACCCAAAGGATTGAGGTCTGTTTTAGAGCTAG<br>AAATAGCAAGTTAAAATAAGGCTAGTCCGTTATCAACTTGAAAAAGTGGCACCGAGTCGGTGC<br>TTTTTTTCTAGACCCAGCTTTCTTGTACAAAGTTGGCATTACTTCGTGCGTTTGGGGTTTC
**ssODN**	CCCTGTATTACGTGGCAGAGAGTTTTAATGATGCCAAGGAGAAAGTAAGGAACTTTGCTGCCA<br>CAATACCTCGGCCCTTCTCAGTTCGCTACGACCCATACACCCAAAGGATTGAGGTCTTGGACAA<br>TACCCAGCAGCTTAAGATTTTGGCTGATTCCATTAACAGTGAAATTGGAATCCTTT

CRISPR RNA-guided *Fok*I nucleases repair a *PAH* variant in a phenylketonuria model

Yi Pan, Nan Shen, Sabine Klawitter-Jung, Christian Betzen, Georg F Hoffmann, Jörg D Hoheisel, Nenad Blau

Supplementary Table 3. Primers used in this work.

**PAH forward**	AGCAAGAATTCATGTCCACTGCGGTCCTG
**PAH reverse**	ATACAGAGCGGCCGCTTACTTTATTTTCTGGAGGGCACT
**EF1α forward**	TCAAGCCTCAGACAGTGGTTC
**IRES reverse**	CCTCACATTGCCAAAAGACG
**M13 reverse**	CAGGAAACAGCTATGAC

CRISPR RNA-guided *Fok*I nucleases repair a *PAH* variant in a phenylketonuria model

Yi Pan, Nan Shen, Sabine Klawitter-Jung, Christian Betzen, Georg F Hoffmann, Jörg D Hoheisel, Nenad Blau

Full-length gels and blots Supplementary Information

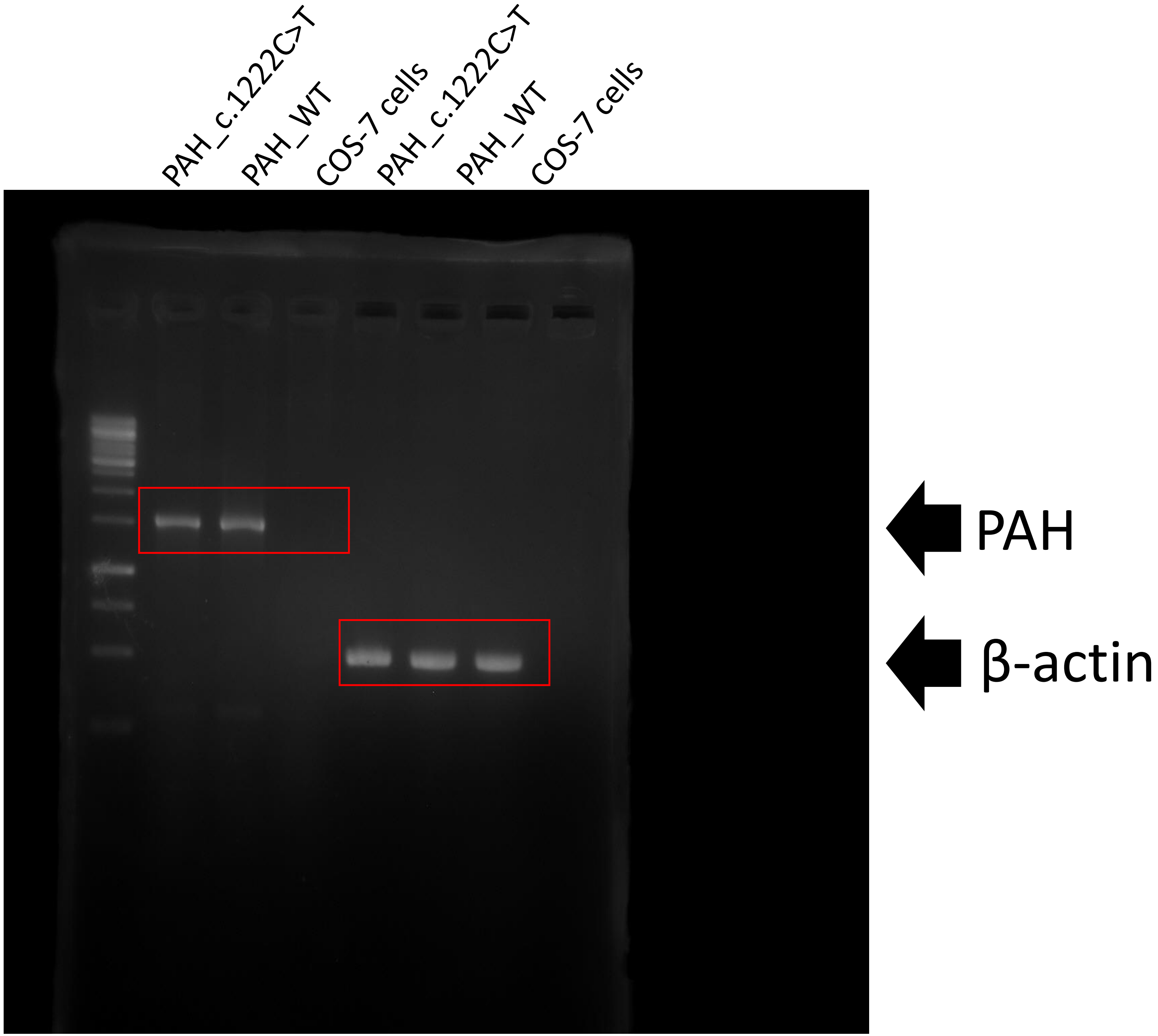
PAH_c.1222C>T
PAH_WT
COS-7 cells
PAH_c.1222C>T
PAH_WT
COS-7 cells
PAH
β-actin

Fig 1b.

Fig 1c. PAH and β-actin Western Blot

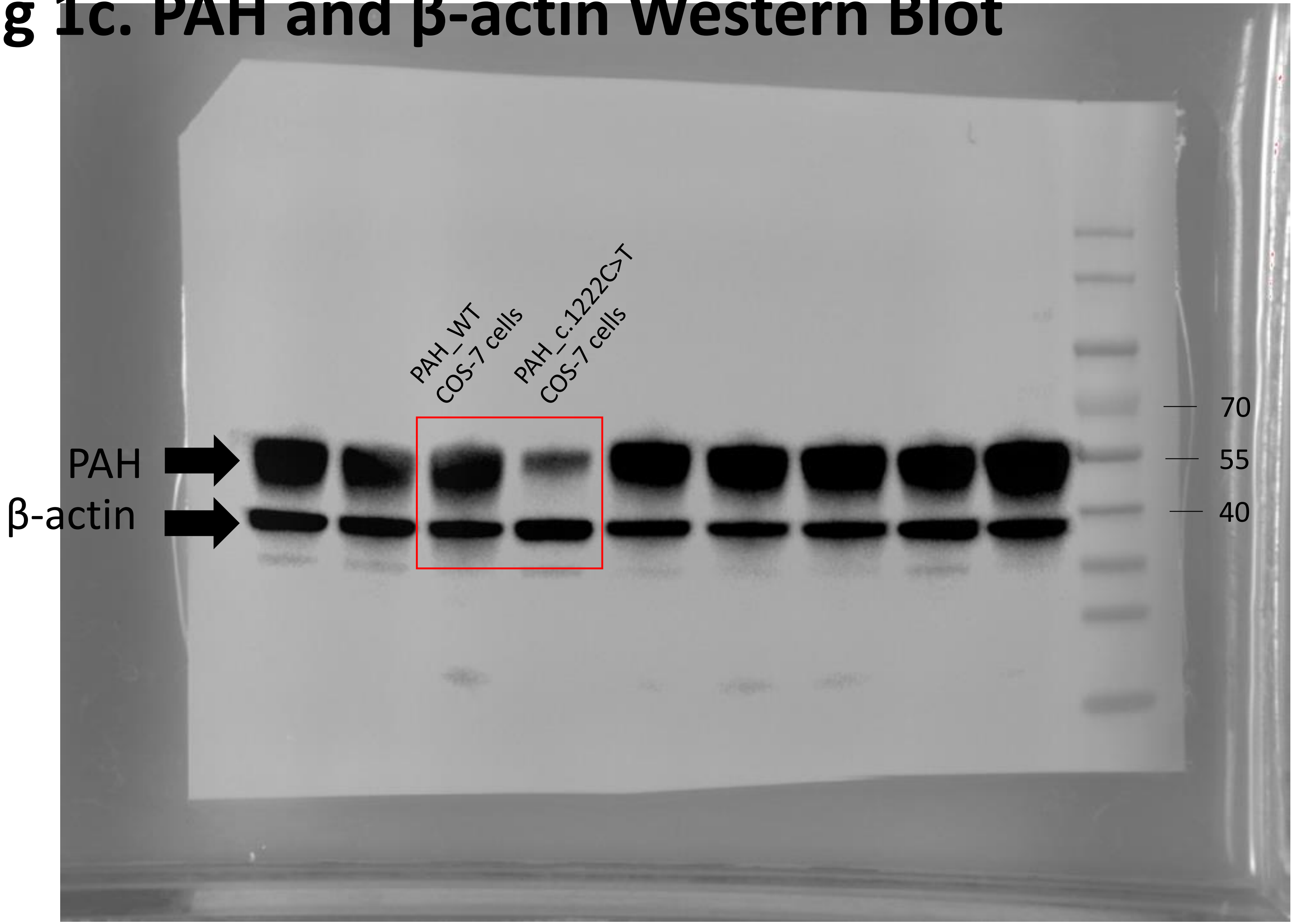

Fig 2c. PAH Western Blot

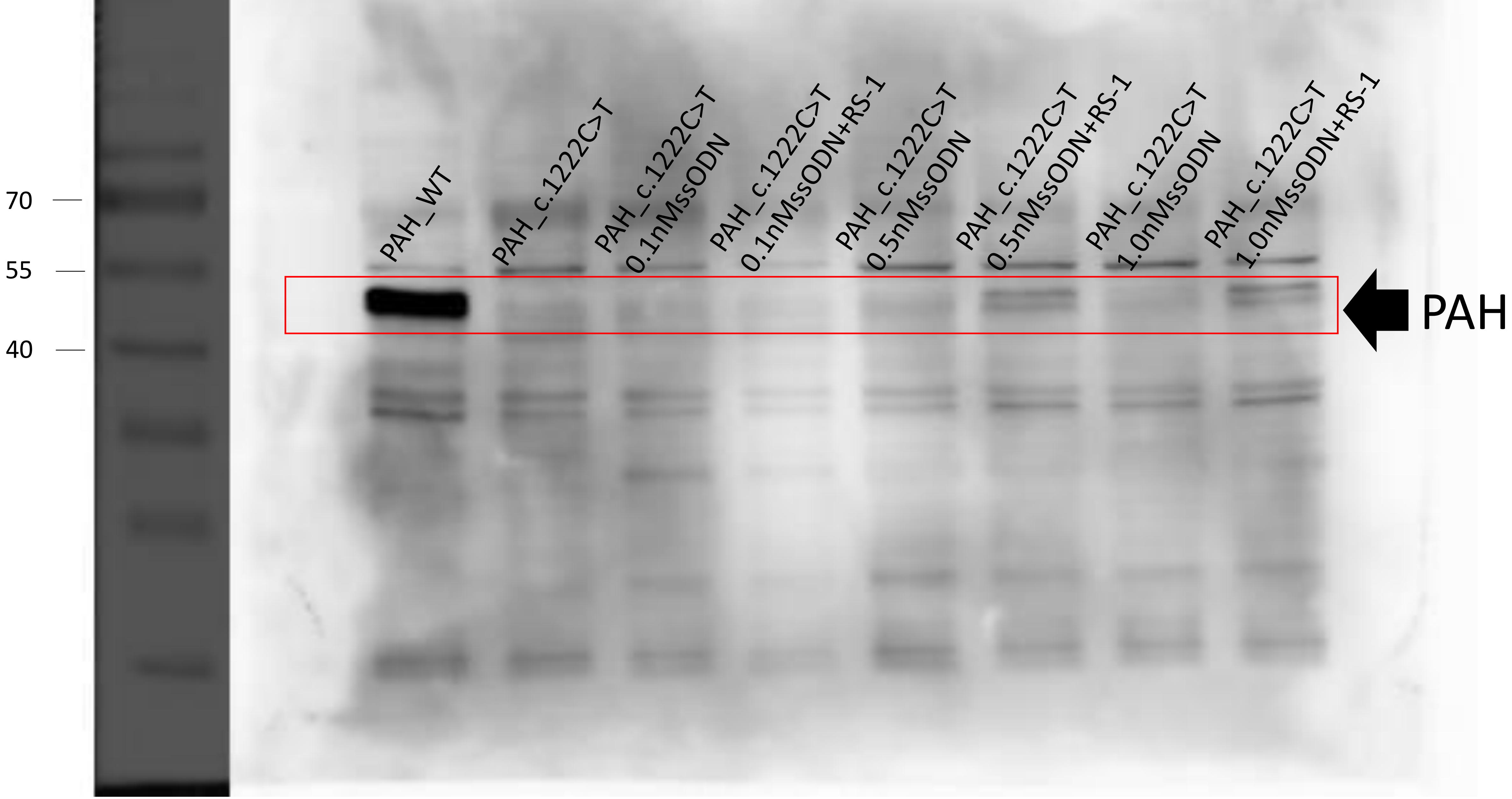

Fig 2c. β-actin Western Blot

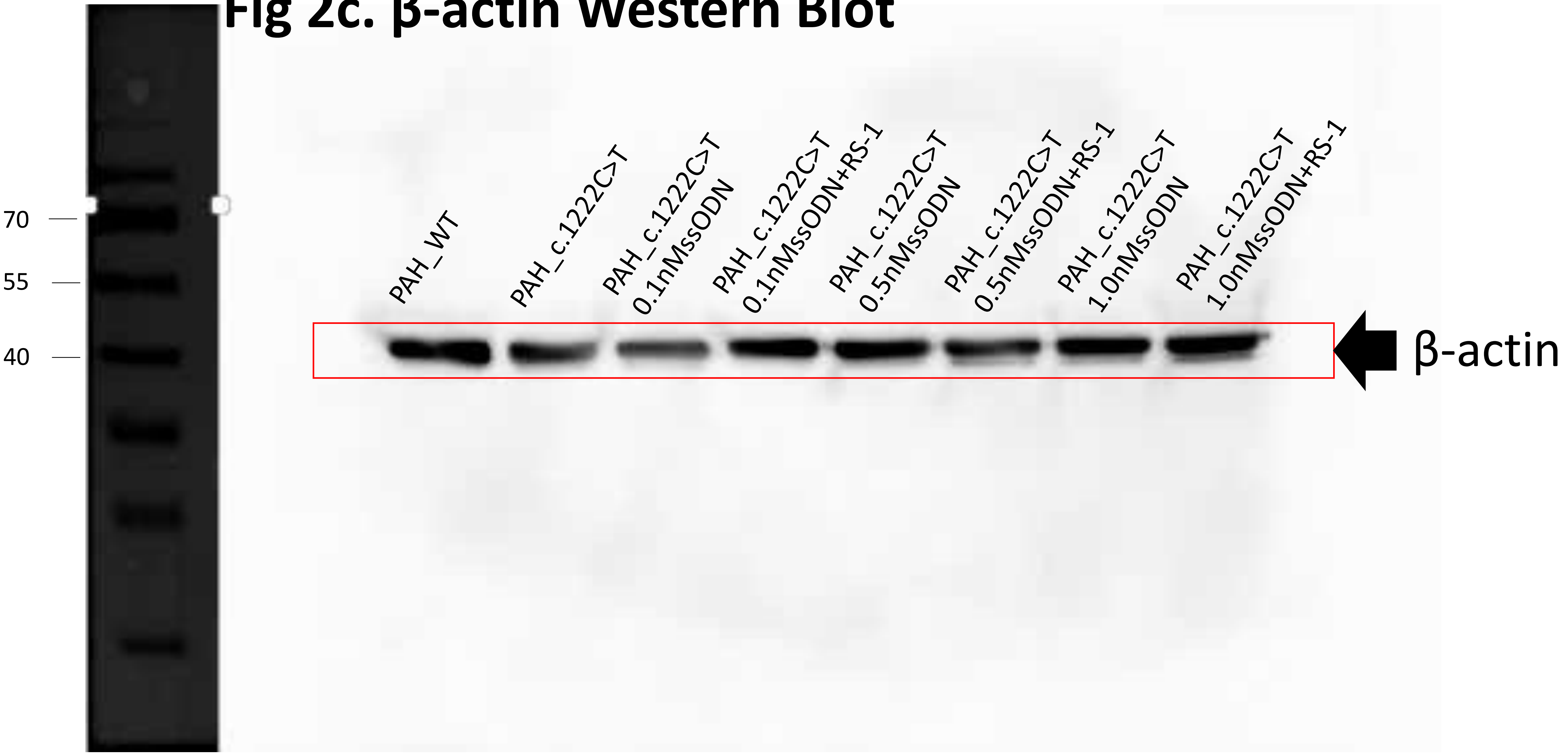

Supplement: Supplementary Information [file srep35794-s1.pdf]
